# Ethnic Disparities in Severe Maternal Morbidity and the Contribution of Deprivation: A Population‐Based Causal Analysis

**DOI:** 10.1111/1471-0528.18254

**Published:** 2025-06-13

**Authors:** Dorothea Geddes‐Barton, Raph Goldacre, Marian Knight, Nicola Vousden, Rema Ramakrishnan

**Affiliations:** ^1^ National Perinatal Epidemiology Unit, Nuffield Department of Population Health University of Oxford Oxford UK; ^2^ Nuffield Department of Population Health Oxford University Oxford UK; ^3^ Oxford NIHR Biomedical Research Centre Oxford UK

**Keywords:** deprivation, ethnicity, health disparities, maternal health, severe maternal morbidity

## Abstract

**Objective:**

To investigate the association between ethnicity and severe maternal morbidity (SMM) in England and the mediating effects of neighbourhood‐level socio‐economic deprivation across detailed ethnic groups.

**Design:**

Population‐based nationwide cohort study using English Hospital Episode Statistics Admitted Patient Care (HES APC) data.

**Setting:**

All hospital births in NHS facilities in England between 1 January 2013 and 31 March 2023.

**Population:**

A cohort of 3 839 156 women aged 10– 55 years with births of ≥ 20 weeks' gestation.

**Methods:**

Multivariable fixed and mixed‐effects Poisson regression models were used to estimate adjusted risk ratios (RR) for SMM across 10 ethnic groups compared to White women and for each ethnic group in different deprivation quintiles compared to White women in the least deprived neighbourhoods, respectively. Causal mediation analysis was used to calculate the proportion of the association mediated by deprivation.

**Main Outcome Measures:**

The modified English Maternal Morbidity Outcome Indicator (EMMOI), a composite outcome of SMM.

**Results:**

Minoritised ethnic groups experienced higher SMM risks than White women, with the highest risk for Black African women (RR 1.96, 95% CI: 1.82–2.02) and Bangladeshi women (RR 1.97, 95% CI: 1.88–2.07) compared to White women. The strength of the association varied across ethnic subgroups. Most of the effect of ethnicity on SMM was not mediated by deprivation (11%–29%).

**Conclusions:**

Deprivation plays a minor role in ethnic disparities in SMM. Policies must address the unique challenges faced by minoritised ethnic women.

## 1 Introduction

In the United Kingdom, individuals from minoritised ethnic groups have an increased risk of adverse maternal health outcomes. The UK Confidential Enquiry into Maternal Deaths has consistently shown that women of Black and Asian backgrounds experience significantly higher maternal mortality rates than White women [1]. However, as maternal mortality is rare in the United Kingdom, data are presented in broad ethnic categories, potentially concealing granular differences across specific ethnic groups.

Maternal mortality is useful for highlighting general disparities and assessing broad trends in maternal health. However, the World Health Organization (WHO) has recommended that severe maternal morbidity (SMM) may be a complementary and potentially more informative measure in high‐income countries where the risk of maternal mortality is, fortunately, very low. SMM, or life‐threatening events that happen in pregnancy and around the time of birth, such as haemorrhage or eclampsia, occur more frequently with an incidence of 1%–2% of all births [2]. SMM not only results in physical and psychological trauma for women [3], but SMM studies can provide quicker and more robust conclusions, offering a larger sample size with a greater power to study the quality of maternal health systems and trends in maternal health [4].

The risk factors for SMM among minoritised ethnic women are complex and thought to be interrelated, including broader structural and cultural factors such as education and employment, access to healthcare services and individual health risks such as obesity or pre‐existing medical conditions [5]. Fundamental cause theory [6] emphasises that health inequalities arise from unequal access to essential resources, such as money, power and social networks, which may be shaped by broader social forces such as racism and residential segregation. This is especially relevant as a disproportionate number of women from minoritised ethnic groups live in the most socio‐economically deprived areas of the United Kingdom [7, 8], areas marked by limited access to income, education, employment and housing, and living in a deprived area itself significantly increases the risk of adverse maternal health outcomes [1, 7, 8].

Since both minoritised ethnicity and neighbourhood‐level deprivation are associated with SMM and strongly associated with each other, there is a need to disentangle their respective impacts on SMM regarding their plausible roles in the causal pathway. Therefore, this study aimed to examine the association between ethnicity and SMM in England, focusing on the gradient effects of deprivation, as well as a potential mediator, on the risk of SMM across disaggregated ethnic groups. The study also aimed to provide a more detailed understanding of the intersection between ethnicity and neighbourhood‐level deprivation and any increased risk of SMM.

## 2 Methods

### 2.1 Study Design

The study was a nationwide retrospective cohort study using the English National Hospital Episode Statistics Admitted Patient Care (HES APC) database.

### 2.2 Data Source

The HES APC is a nationwide administrative database that records all hospital admissions across the NHS in England, including 97% of births [9]. The HES APC contains demographic and clinical information, including an additional maternity section containing pregnancy and birth‐specific information. All childbirth episodes from 1 January 2013 to 31 March 2023 were extracted, and all hospital admissions from 2003 were subsequently linked. Diagnoses were coded using the 10th edition of the International Classification of Diseases (ICD‐10), and operative procedures using the Office for Population Censuses and Surveys Classification, 4th revision (OPCS‐4).

### 2.3 Ethics Committee Approval, Data Availability and Reporting

Under the assessment of the NHS Health Research Authority, using the HES APC data to conduct epidemiological and health service research at the University of Oxford does not need research ethics committee approval as it is anonymised data. This study is reported according to recommendations in the RECORD Guidelines [10].

### 2.4 Study Population, Exposure and Outcome (See Supplementary Methods Section for More Details)

This study included all women aged 10–55 who gave birth (including single and multiple pregnancies) between 1 January 2013 and 31 March 2023 in a hospital, with gestational age at childbirth ≥ 20 weeks, and complete data on ethnicity and deprivation [11]. If a woman had more than one maternity in the time period, only one was randomly selected to eliminate the effect of clustering within women over time. Ethnicity was defined using the Office for National Statistics categorisation system [12] collapsed into 10 groups based on the categories used for the MBRRACE‐UK perinatal mortality surveillance report [13]: White, Black or Black British African, Black or Black British Caribbean, Asian or Asian British Indian, Asian or Asian British Pakistani, Asian or Asian British Bangladeshi, Mixed, Other, Other Black and Other Asian. Neighbourhood deprivation was defined based on the Index of Multiple Deprivation (IMD), which is a composite measure that consists of seven domains measured at neighbourhood level including income, employment, education, crime, living environment, barriers to services and health, each reflecting aspects of deprivation within neighbourhoods of around 1500 residents. An overall IMD score for each neighbourhood is calculated as a weighted sum of the ranks in each domain, and these scores are ranked at a national level. The outcomes included a composite of SMM diagnoses and procedures, defined using the modified English Maternal Morbidity Outcome Indicator (EMMOI) [10] Table S1.

### 2.5 Statistical Analysis

Statistical analysis was performed using Stata version 18. Statistical significance was assumed to be a *p* value of less than 0.05. The incidence of SMM in each ethnic group is presented using the number of maternities (women with either live or stillbirths) as the denominator. The characteristics of the women are presented as numbers and percentages in each group stratified by ethnicity. Fixed‐effects multivariable Poisson regression models were built to estimate the risk ratios and associated 95% confidence intervals (CI) of SMM for each ethnic group compared to White women using a complete case analysis. Models were adjusted for 1) age and year of birth; 2) Model 1 + parity.

To estimate the combined effect of ethnicity and deprivation, multivariable, mixed‐effects Poisson regression models with a random intercept were used to calculate the incidence rate (and 95% CIs) to account for clustering between individuals who shared the same postcode (LSOA), for each ethnic group and IMD quintile compared to White women living in the least deprived quintile, adjusted for age, parity and year of childbirth. Level 1 was defined as the individual woman and Level 2, her LSOA. The model with a random intercept had a better fit than the fixed‐effects model (likelihood ratio test: *p* < 0.001). The intra‐class coefficient (ICC) was used to quantify the proportion of variance that can be attributed to the LSOAs. Average adjusted predictive margins and their 95% CI for each IMD quintile and ethnic group were then calculated to quantify the absolute risks of SMM in each ethnic and IMD subgroup, adjusting for age, parity and year of childbirth.

### 2.6 Mediation

The mediation analysis was conducted in a subsample of the women residing in the most and least deprived IMD quintiles. A directed acyclic graph (DAG) (Figure S1) was used to select potential confounders for mediators based on both existing literature and clinical knowledge a priori. The confounders included age, year of birth and parity. The *mediate* [14] command in Stata was used to examine the mediating effects of deprivation on SMM, where an exposure–mediator interaction was specified for the final model, as the results differed when this was included in the model compared to when it was excluded. The mediation results presented are the total effect (TE), natural indirect effect (NIE), natural direct effect (NDE) controlled direct effect (CDE), and proportion mediated (Table S2). The TE quantifies the overall relationship between the exposure and the outcome irrespective of mediator status; NIE quantifies the portion of this relationship mediated after accounting for exposure–mediator interaction; NDE quantifies the effect of the exposure on the outcome that is not mediated; and CDE quantifies the direct effect when the mediator is fixed at a certain value. A Poisson regression model was used for the outcome, and a logistic regression model was used for the mediator to estimate risk and odds ratios respectively. The exposure was each minoritised ethnic group compared to White women as the reference group. The mediator was a binary variable, comparing women living in the most deprived IMD quintile to women living in the least deprived IMD quintile.

## 3 Results

### 3.1 Characteristics of the Study Population

The characteristics of the women in each ethnic group are shown in Table S3. For the final study population of 3 839 156 women **(**Figure S2) with a mean age at childbirth of 30.4 years, the overall risk of SMM was 1.75% (Table S3). A greater proportion of Black Caribbean (44.2%), Black African (47.3%), Pakistani (51.3%), Bangladeshi (52.0%) and Other Black (42.7%) women lived in the most deprived IMD quintile compared to the average in the cohort (21.6%). The number and proportion of women with SMM in each ethnic group by deprivation quintile are shown in Table S4.

### 3.2 Multivariable Analysis

The risk ratios and their 95% CIs for the sequential models are shown in Table 1. Age was modelled as a categorical variable as there was significant evidence for departure from linearity (*p* < 0.001). Compared to White women, the risk of SMM was higher for all minoritised ethnic groups, with Black African women, Other Black, and Bangladeshi women having the highest risk, with risk ratios of 1.88 (95% CI 1.82–1.95), 1.79 (95% CI 1.68–1.90) and 1.83 (95% CI 1.74–1.92), respectively, compared to White women. After additional adjustment for parity (Table 1, Model 3), the risk ratios increased to 1.96 (95% CI 1.89–2.02), 1.85 (95% CI 1.74–1.96) and 1.97 (95% CI 1.88–2.07), respectively.

**TABLE 1:** The association between ethnicity and severe maternal morbidity.

	Total Number of women	Number of women with SMM	Model 1: adjusted for age and year of birth	Model 2: adjusted for age, parity, and year of birth
	N [%]	N [%]	RR [95% CI]	RR [95% CI]
White	2 939 689 (76.6)	45 889 [1.6]	1 [ref]	1 [ref]
Black or Black British—Caribbean	37 046 (1.0)	978 [2.6]	1.68 [1.58–1.79]	1.74 [1.63–1.85]
Black or Black British—African	119 491 (3.1)	3596 [3.0]	1.88 [1.82–1.95]	1.96 [1.89–2.02]
Asian or Asian British—Indian	135 491 (3.5)	2833 [2.1]	1.29 [1.25–1.35]	1.29 [1.24–1.34]
Asian or Asian British—Pakistani	149 880 (3.9)	3423 [2.3]	1.46 [1.41–1.51]	1.53 [1.48–1.59]
Asian or Asian British—Bangladeshi	56 646(1.5)	1653 [2.9]	1.83 [1.74–1.92]	1.97 [1.88–2.07]
Mixed	74 342 (1.9)	1488 [2.0]	1.21 [1.15–1.27]	1.22 [1.15–1.28]
Other	190 615 (5.0)	4114 [2.2]	1.34 [1.29–1.38]	1.32 [1.28–1.37]
Other Asian	99 498 (2.6)	2507 [2.5]	1.54 [1.48–1.61]	1.55 [1.48–1.61]
Other Black	36 458 (0.9)	1033 [2.8]	1.79 [1.68–1.90]	1.85 [1.74–1.96]

Abbreviations: IMD = index of multiple deprivation, RR = risk ratio.

**FIGURE 1:**
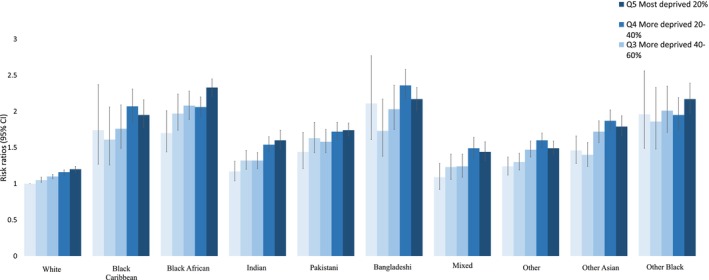
Risk ratios (and their 95% confidence intervals) of SMM adjusted for age, parity and year of childbirth for each ethnic group and IMD quintile compared to White women living in the least deprived IMD quintile.

In the mixed‐effects model that examined the combined effect of ethnicity and deprivation, there were 33 977 neighbourhoods modelled at the LSOA level. The maternities per neighbourhood ranged between 1 and 1001, with an average of 120. Neighbourhood differences accounted for minimal variation in SMM with an ICC of 3.8% (95% CI 3.1%–4.1%) (Level 2) and the remaining 96.2% (95% CI 96.0%–96.5%) was due to the variance between individuals within neighbourhoods (Level 1). The adjusted risk ratios and their 95% CI for SMM of each ethnic group and IMD quintile compared to White women in the least deprived quintile, adjusted for age, parity and year of childbirth, are shown in Figure 1. There was a dose–response relationship seen between deprivation and SMM for White women. Broad gradient effects of IMD were observed across all ethnic groups. There were elevated rates of SMM in many ethnic groups such that even women in the least deprived quintiles experienced higher rates of SMM than the most deprived White women. The average adjusted predictive margins and their 95% CI are shown in Table S5.

### 3.3 Mediation

**FIGURE 2:**
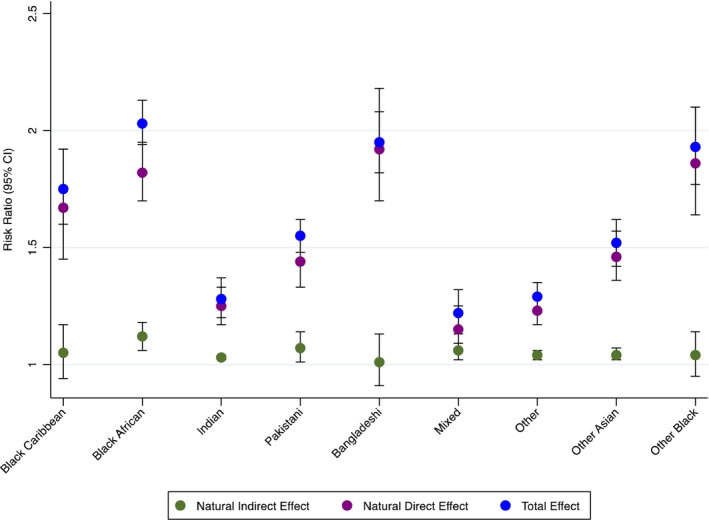
Effects plot for the effect of ethnicity on SMM mediated by deprivation, adjusting for age, parity and year of childbirth.

Women from minoritised ethnicity had higher risk for SMM, irrespective of mediator status. The adjusted risk ratios ranged from 1.22 (95% CI: 1.13–1.32) for women of Mixed ethnicity to 2.03 (95% CI: 1.94–2.13) for Black African women, compared to White women (Table 2). The NDE was consistently larger than the NIE, showing that while deprivation may partially mediate this association, being from a minoritised ethnicity alone is a more important risk factor. For ethnic groups with significant mediation effect, the proportion of the total effect of ethnicity mediated by deprivation after accounting for exposure–mediator interaction varied from 29% (95% CI: 10%–49%) in women of Mixed ethnicity to 11% (95% CI: 4%–18%) for women of ‘Other Asian’ ethnicity compared to White women. The effects plot of the TE, NDE and NIE is shown in Figure 2. The effects of ethnicity controlling for two fixed levels of the mediator (CDE) are shown in Table S6.

**TABLE 2:** Results from the mediation analyses for the association between ethnicity and SMM with deprivation as mediator, adjusting for age and parity and year of childbirth.

Ethnicity	NIE (RR [95% CI])	NDE (RR [95% CI])	TE (RR [95% CI])	Proportion mediated [95% CI]
Black or Black British—Caribbean	1.05 [0.94, 1.17]	1.67 [1.45, 1.92]	1.75 [1.60, 1.92]	0.11 [−0.13, 0.35]
Black or Black British—African	1.12 [1.06, 1.18]	1.82 [1.70, 1.95]	2.03 [1.94, 2.13]	0.21 [0.11, 0.30]
Asian or Asian British—Indian	1.03 [1.02, 1.04]	1.25 [1.17, 1.33]	1.28 [1.20, 1.37]	0.14 [0.08, 0.20]
Asian or Asian British—Pakistani	1.07 [1.01, 1.14]	1.44 [1.33, 1.55]	1.55 [1.48, 1.62]	0.20 [0.04, 0.35]
Asian or Asian British—Bangladeshi	1.01 [0.91, 1.13]	1.92 [1.70, 2.18]	1.95 [1.82, 2.08]	0.03 [−0.19, 0.24]
Mixed	1.06 [1.02, 1.09]	1.15 [1.06, 1.25]	1.22 [1.13, 1.32]	0.29 [0.10, 0.49]
Other	1.04 [1.02, 1.06]	1.23 [1.17, 1.30]	1.29 [1.22, 1.35]	0.18 [0.08, 0.27]
Other Asian	1.04 [1.02, 1.07]	1.46 [1.36, 1.57]	1.52 [1.42, 1.62]	0.11 [0.04, 0.18]
Other Black	1.04 [0.95, 1.14]	1.86 [1.64, 2.10]	1.93 [1.77, 2.10]	0.08 [−0.10, 0.27]

*Note:* Results from model that included exposure‐mediator interaction.

Abbreviations: CI= confidence interval, NDE= natural direct effect, NIE= natural indirect effect, RR= risk ratio, TE= total effect.

## 4 Main Findings

This study of 3 839 156 births in England from 1 January 2013 to 31 March 2023 showed an overall incidence of SMM of 1.8%. Minoritised ethnic women were disproportionately represented in the most deprived areas, with Black (African, Caribbean and Other Black) and Asian (Pakistani and Bangladeshi) women most likely to reside in the most deprived IMD quintile and more likely to experience SMM.

Black African and Bangladeshi women were at the highest overall risk of SMM. The probability of SMM was greatest for women living in the fourth and fifth most deprived quintiles of areas compared to the least deprived areas in each of the ethnic groups. Most of the association between women from minoritised ethnic groups compared to White women and SMM was not mediated by deprivation. However, small numbers in some minoritised ethnic groups (Black Caribbean, Other Black, and Bangladeshi) residing in the least deprived areas limited the ability to effectively study the mediation effects in these populations.

## 5 Strengths and Limitations

To our knowledge, this is the first study in the United Kingdom to examine neighbourhood deprivation as a mediator in the relationship between ethnicity and SMM. A key strength of this study is the large dataset, which included 97% of births in England over a 10‐year period, which minimises the risk of selection bias and increases the generalisability of the findings. Additionally, the large sample size enabled us to conduct disaggregated analysis by ethnicity.

However, as the data are sourced from HES APC rather than data collected for research purposes, there is missing information on important confounders such as parity and the risk of false‐negatives of the outcome coded using ICD‐10 and OPCS‐4 codes. Parity is more likely to be under‐recorded for women who recently migrated to England and will not have had any care outside of the NHS recorded in the database. Parous women are at a lower risk of SMM [15], which may lead to differential unmeasured confounding. Therefore, the association between ethnicity and SMM may be underestimated for migrant women if they are incorrectly classified as nulliparous. The small numbers in certain ethnic groups living in the least deprived areas may have resulted in insufficient power to detect an association and could explain the uncertainty in some of the estimates. Though the assumption of temporality of the exposure, mediator and outcome was not violated and we can assume no unmeasured confounding for ethnicity–deprivation and ethnicity– SMM, we were unable to test two of the remaining assumptions [16] because of residual confounding.

## 6 Interpretation

In this study, Black African, Other Black and Bangladeshi women experienced the highest overall risk of SMM compared to White women. This is consistent with findings from a case–control study in the United Kingdom conducted by Nair et al. [17]. The results also align with that of the UK maternal mortality surveillance report for 2021–2023 [1], which showed that Black women are nearly four times and Asian women twice as likely to die from pregnancy‐related complications than White women. However, our study revealed a difference in the strength of the association among specific ethnic subgroups, notably showing that Bangladeshi and Black African women face a significantly higher risk of SMM. Therefore, combining all Asian or Black women into a single group would likely misrepresent the association with SMM. These findings highlight the pitfalls of using broad ethnic groups for the analysis as they may conceal the differences in life course exposure to opportunity, adversity and discrimination, patterned by structural systems of oppression, immigration patterns and policies [18], experienced by different ethnic subgroups.

This study did not find a clear dose–response relationship between increasing deprivation and SMM risk in some minoritised ethnic groups. Vousden et al. reported a similar pattern for odds of maternal mortality among women of Black and Asian ethnic minority [7]. However, these results are in contrast with findings from a US study that reported a dose–response relationship of poverty with SMM among Black Latina women in New York City [19]. Moreover, in this study, except for Mixed and Indian ethnicity women, all minoritised ethnic women living in the least deprived quintile had a greater risk of SMM than White women living in the most deprived quintile. This was also found in Vousden's study [7] for Black but not Asian women and risk of maternal mortality. The absence of a consistent gradient in minoritised ethnic groups adds evidence to the argument that policy interventions should extend beyond the most deprived populations to address disparities across the entire population. Adopting a strategy of proportional universalism, where interventions are applied universally but with intensity proportionate to need, may help ensure that support reaches all groups equitably while addressing the structural and systemic factors contributing to maternal health inequalities [20]. However, these findings should be interpreted with caution due to uncertainty in some estimates for women from minoritised ethnic groups living in the less deprived areas.

Living in a deprived area did not play a substantial role in the association between ethnicity and SMM. For Bangladeshi, Black Caribbean and Other Black women, there was no evidence of mediation by deprivation. However, these groups had small numbers of women living in the least deprived areas, and therefore, there was likely limited statistical power to detect mediation. For the remaining minoritised ethnic groups, the proportion mediated varied from 11% to 29%, revealing that living in a deprived area may contribute to, but does not fully explain the increased risk associated with ethnicity. It is unclear from this study what other factors are driving the increased risk, though systemic, institutional and interpersonal racism, and disparities in access to healthcare services and quality of care may be contributors to the disparities observed [5]. Moreover, the IMD as an area‐based measure may not capture individual socio‐economic position or all the unique individual economic and structural inequalities experienced by minoritised ethnic groups. Minoritised ethnic women with high socio‐economic position may reside in deprived areas due to factors such as migration patterns, housing discrimination or ethnic density which may result in misclassification if the IMD is taken as a proxy for individual socio‐economic position. Therefore, alternative measures of socio‐economic position, including individual measures, could play a more significant role.

## 7 Conclusion

This study highlights the importance of analysing maternal outcomes such as SMM using disaggregated ethnic subgroups. Although all minoritised ethnic women had greater risks of SMM compared to White women, Black African and Bangladeshi women faced a particularly high risk, with neighbourhood deprivation only playing a minor role. These findings underscore the need for research and policies that address not only social, economic, and environmental factors but also other underlying causes of persistent risks faced by minoritised ethnic women. Additionally, future research is needed to examine the role of various socio‐economic disadvantage measures on the association between ethnicity and SMM and maternal mortality, including individual metrics, to more adequately reflect the lived experiences of diverse populations.

## Author Contributions

D.G.‐B. conceptualised the project and undertook the literature review with support from N.V., R.R. and R.G. D.G.‐B., R.R., M.K., N.V. and R.G. contributed to study design. D.G.‐B. and R.R. analysed the data and all authors contributed to data interpretation and writing. All authors accept responsibility for the paper as published.

## Disclosure

The authors have nothing to report.

## Ethics Statement

Under the assessment of the NHS Health Research Authority, using the HES APC data to conduct epidemiological and health service research at the University of Oxford does not need research ethics committee approval as it is anonymised data.

## Conflicts of Interest

The authors declare no conflicts of interest.

## Supporting information


**Data S1** Supporting Information

## Data Availability

Data may be obtained from a third party and are not publicly available. The data extract was derived from the English National Hospital Episode Statistics Admitted Patient Care (HES‐APC) database with linkage to national mortality civil registrations (https://digital.nhs.uk/data‐and‐information/data‐tools‐and‐services/dataservices/linked‐hes‐ons‐mortality‐data). Linked HES‐APC and mortality data are available upon application to NHS England (formerly NHS Digital).
