# Supplementary material for: Ethnic Disparities in Severe Maternal Morbidity and the Contribution of Deprivation: A Population‐Based Causal Analysis
Source: BJOG. 2025 Jun 13;132(13):2131–7. doi: 10.1111/1471-0528.18254 (PMC12592783; doi:10.1111/1471-0528.18254)
Supplement: Supplementary file 1 — Data S1 Supporting Information [file BJO-132-2131-s001.docx]

**Supplementary material**

**Supplementary Methods**

**Data source**

The Hospital Episode Statistics Admitted Patient Care (HES APC) is a routinely collected dataset that captures clinical and administrative information for patients discharged from hospital care. When a patient is discharged, the attending clinician completes a discharge summary, which includes details of diagnoses and procedures performed during the hospital stay. This information, along with demographic and administrative data, is entered into a local electronic database by trained clinical coders. National Health Service (NHS) Digital (which was succeeded by NHS England in February 2023) processes and validates the data. Although the HES APC was not initially designed for research purposes, it is widely used in research and service evaluation due to its ability to track individuals over time, its broad coverage, and its extensive data collection period. The dataset includes pregnancy and birth-specific details, such as the mode of birth, birth weight, gestational age at childbirth, maternal age, the attending clinician, and the birth location, stored in a separate maternity section. However, submission of this maternity data is not mandatory for all hospitals, resulting in variability in data completeness and quality across different NHS hospitals (1).

**Creating the cohort**

The cohort was created using delivery "superspells" from 1st January 2013 to 31st March 2023. A "spell" refers to a continuous period of hospitalisation under a single consultant, which can include multiple "episodes" of care. A "superspell" extends this period of care to include transfers between NHS hospitals. Each patient is assigned a unique encrypted ID, which is used to link all episodes of care for the same individual. Information on confounding factors from 2003 was added to each row using a ‘look back’ approach. This method involved flagging records with pre-selected diagnosis or procedure codes and copying the relevant dates from the first instance of each condition onto all corresponding “superspells” with matching encrypted IDs. Finally, any completed “superspells” that were not related to childbirth (i.e., those without OPCS-4 codes R17–R25) were excluded to form the final birth cohort.

**Duplicate ‘superspells’**

Exact duplicate ‘superspells’ were dropped. If information was missing on one but present on a duplicated ‘superspell’ ID, the information was copied over and the duplicated ‘superspells’ dropped. If a duplicated ‘superspell’ had conflicting gestational age at childbirth, birthweight or maternal age both records were dropped.

**Defining Gestational Age at Birth**

Only birth ‘superspells’ which resulted in a pregnancy loss, live or stillbirth ≥ 20 weeks gestational age at childbirth were included. Gestational age at childbirth and birthweight of the baby were used to determine a cohort of a gestational age at childbirth ≥ 20 weeks. Data was set to missing if it was thought to contain implausible values such as if gestational age at childbirth was > 45 weeks. Birthweight was recoded as implausible if it was greater than 5 SD away from the median for the corresponding gestational age. However, in the current HES APC dataset there was 25.0% missing information on gestational age at childbirth. If data on gestational age at childbirth was missing, the gestational age at childbirth was assumed to be ≥ 20 weeks if birthweight was recorded and was > 360g which is the median birthweight for 20 weeks. There was 13.1% missing data on both birthweight and gestational age at childbirth. These women were included in the cohort as the percentage of women with a gestational age at childbirth < 20 weeks was 0.5% in the women with complete data. Therefore, the number of women with a missing value of gestational age at childbirth, who were < 20 weeks’ gestation are likely to be low.

**Study Population**

This study included all women aged 10 to 55 who gave birth (including singleton and multiple pregnancies) in a hospital setting between 1st January 2013 and 31st March 2023, with a gestational age at childbirth ≥ 20 weeks and with complete data on Index of Multiple Deprivation (IMD) and ethnicity. Data from all NHS hospitals in England were included in the analysis. Home births, which represent approximately 2.3% of births in England (2), were not included in the dataset.

**Exposure**

Ethnicity was defined using the Office for National Statistics (ONS) categorisation system (3) collapsed into ten groups based on the categories used for the MBRRACE-UK perinatal mortality surveillance report (4): White, Black or Black British African, Black or Black British Caribbean, Asian or Asian British Indian, Asian or Asian British Pakistani, Asian or Asian British Bangladeshi, Mixed, Other, Other Black and Other Asian. The Office for National Statistics compared individual ethnicity data recorded in HES with self-reported ethnicity data from the 2011 census, which is considered the gold standard (5). Of the total population in the 2011 census, 66% could be both linked to HES and had recorded ethnicity information. Among these, there was a 90.7% match in ethnicity when categorised into 18 ethnic groups, and a 95.8% match when grouped into 5 broad ethnic categories between the two sources. There was 14.98% missing data on ethnicity. This was reduced to 4.98% by copying ethnicity from any hospital admission in the database prior to creating the final cohort of just birth episodes.

**Outcome**

**Severe maternal morbidity (SMM)**

SMM was defined using a modified English Maternal Morbidity Outcome Indicator (EMMOI) (6). This is a composite outcome which includes 21 diagnoses and 16 procedures, adapted from the original EMMOI (7), which can be used as a single measure of severe morbidity during childbirth using data from HES APC. The list of the relevant diagnoses/ procedures and their codes are included in the **Table S1**.

**Covariates**

Covariates included the following: maternal age at childbirth, neighbourhood deprivation, year of childbirth and parity.

**Maternal age at childbirth**

Maternal age at childbirth was grouped into five-year categories except for ages younger than 20 or older than 40+ which were grouped into single categories.

**Neighbourhood deprivation**

Neighbourhood deprivation defined based on the 2015 and 2019 release of the IMD. For births occurring before January 1, 2015, the 2015 IMD release was applied, whereas for births on or after January 1, 2015, the 2019 IMD release was used. In this study, national rankings were divided into quintiles, with the first quintile representing the least deprived areas and the fifth representing the most deprived. Each woman was linked to a neighbourhood based on her postcode at the time of birth, which allowed assignment to a specific deprivation quintile. For the causal mediation analysis, the most deprived quintile was compared with the least deprived quintile.

**Parity**

The HES APC dataset contains a substantial amount of missing data on parity (35.7%). An algorithm was applied across the dataset to address inconsistencies in parity data and to estimate parity when it was missing. First, if any healthcare trust reported an implausible proportion of nulliparous women (outside the range of 20–70%), all parity data from that trust was set to ‘unknown’. Parity was then assigned based on the value in the NUMPREG field and a ‘look-back’ method.

A woman was classified as multiparous if her parity was greater than 0 or missing in the NUMPREG field, if there was evidence of a previous delivery code (R17-R25) from 2003, if a code suggesting a previous delivery (such as previous caesarean section) appeared during the current pregnancy (codes O34.2, O75.7, Z35.4, Z87.6, and Z87.5), or if it was not her first delivery ‘superspell’ in the database. If none of these criteria flagged a woman as multiparous, she was assumed to be primiparous.

**Table S1**. **Morbidity categories codes used in final composite outcome indicator used to identify events consistent with severe maternal morbidity (modified from the EMMOI) (6)**

| **Morbid event category (ICD-10 codes)** | **Codes** |
| --- | --- |
| Acute abdomen | N73.3 Female acute pelvic peritonitis  N73.5 Female pelvic peritonitis, unspecified  K65.0 Acute peritonitis  K65.9 Peritonitis, unspecified  K35 Acute appendicitis  K35.2 Acute appendicitis with generalized peritonitis  K35.3 Acute appendicitis with localized peritonitis  K35.8 Acute appendicitis, other and unspecified  K37 Unspecified appendicitis  K56.2 Volvulus  K56.5 Intestinal adhesions [bands] with obstruction  K56.6 Other and unspecified intestinal obstruction  K59.3 Megacolon, not elsewhere classified |
| Acute renal failure | O90.4 Postpartum acute renal failure  N17 Acute renal failure  N17.0 Acute renal failure with tubular necrosis  N17.1 Acute renal failure with acute cortical necrosis  N17.2 Acute renal failure with medullary necrosis  N17.8 Other acute renal failure  N17.9 Acute renal failure, unspecified  N19 Unspecified kidney failure  N99.0 Postprocedural renal failure  I12.0 Hypertensive renal disease with renal failure  I13.1 Hypertensive heart and renal disease with renal failure |
| Acute psychosis | F23 Acute and transient psychotic disorders  F23.0 Acute polymorphic psychotic disorder without symptoms of schizophrenia  F23.1 Acute polymorphic psychotic disorder with symptoms of schizophrenia  F23.2 Acute schizophrenia-like psychotic disorder  F23.8 Other acute and transient psychotic disorders  F23.9 Acute and transient psychotic disorder, unspecified  F53.1 Severe mental and behavioural disorders associated with the puerperium, not elsewhere classified |
| Acute cardiac event (cardiac infarction, cardiac failure, cardiomyopathy and cardiac arrest) | O90.3 Cardiomyopathy in the puerperium  I21 Acute myocardial infarction  I21.0 Acute transmural myocardial infarction of anterior wall  I21.1 Acute transmural myocardial infarction of inferior wall  I21.2 Acute transmural myocardial infarction of other sites  I21.3 Acute transmural myocardial infarction of unspecified site  I21.4 Acute subendocardial myocardial infarction  I21.9 Acute myocardial infarction, unspecified  I42 Cardiomyopathy  I42.0 Dilated cardiomyopathy  I42.1 Obstructive hypertrophic cardiomyopathy  I42.2 Other hypertrophic cardiomyopathy  I42.3 Endomyocardial (eosinophilic) disease  I42.4 Endocardial fibroelastosis  I42.5 Other restrictive cardiomyopathy  I42.6 Alcoholic cardiomyopathy  I42.7 Cardiomyopathy due to drugs and other external agents  I42.8 Other cardiomyopathies  I42.9 Cardiomyopathy, unspecified  I43* Cardiomyopathy in diseases classified elsewhere  I43.0* Cardiomyopathy in infectious and parasitic diseases classified elsewhere  I43.1* Cardiomyopathy in metabolic diseases  I43.2* Cardiomyopathy in nutritional diseases  I43.8* Cardiomyopathy in other diseases classified elsewhere  I46 Cardiac arrest  I46.0 Cardiac arrest with successful resuscitation  I46.1 Sudden cardiac death, so described  I46.9 Cardiac arrest, unspecified  I50 Heart failure  I50.0 Congestive heart failure  I50.1 Left ventricular failure  I50.9 Heart failure, unspecified  I11.0 Hypertensive heart disease with (congestive) heart failure  I11.9 Hypertensive heart disease without (congestive) heart failure  I13.0 Hypertensive heart and renal disease with (congestive) heart failure  I13.2 Hypertensive heart and renal disease with both (congestive) heart failure and renal failure |
| Acute respiratory compromise | J81 Pulmonary oedema  J80 Adult respiratory distress syndrome |
| Cerebral oedema or coma | G93.6 Cerebral oedema  R40.2 Coma, unspecified |
| Disseminated intravascular coagulopathy | O45.0 Premature separation of placenta with coagulation defect (including placental abruption with excessive haemorrhage associated with afibrinoginaemia, disseminated intravascular coagulation, hyperfibrinolysis, hyperfibrinogenaemia)  O46.0 Antepartum haemorrhage with coagulation defect (antepartum haemorrhage (excessive) associated with afibrinoginaemia, disseminated intravascular coagulation, hyperfibrinolysis, hyperfibrinogenaemia)  O67.0 Intrapartum haemorrhage with coagulation defect (intrapartum haemorrhage (excessive) associated with afibrinoginaemia, disseminated intravascular coagulation, hyperfibrinolysis, hyperfibrinogenaemia)  D65 Disseminated intravascular coagulation [defibrination syndrome] |
| Cerebrovascular accident | I60 Subarachnoid haemorrhage  I60.0 Subarachnoid haemorrhage from carotid siphon and bifurcation  I60.1 Subarachnoid haemorrhage from middle cerebral artery  I60.2 Subarachnoid haemorrhage from anterior communicating artery  I60.3 Subarachnoid haemorrhage from posterior communicating artery  I60.4 Subarachnoid haemorrhage from basilar artery  I60.5 Subarachnoid haemorrhage from vertebral artery  I60.6 Subarachnoid haemorrhage from other intracranial arteries  I60.7 Subarachnoid haemorrhage from intracranial artery, unspecified  I60.8 Other subarachnoid haemorrhage  I60.9 Subarachnoid haemorrhage, unspecified  I61 Intracerebral haemorrhage  I61.0 Intracerebral haemorrhage in hemisphere, subcortical  I61.1 Intracerebral haemorrhage in hemisphere, cortical  I61.2 Intracerebral haemorrhage in hemisphere, unspecified  I61.3 Intracerebral haemorrhage in brain stem  I61.4 Intracerebral haemorrhage in cerebellum  I61.5 Intracerebral haemorrhage, intraventricular  I61.6 Intracerebral haemorrhage, multiple localized  I61.8 Other intracerebral haemorrhage  I61.9 Intracerebral haemorrhage, unspecified  I62 Other nontraumatic intracranial haemorrhage  I62.0 Subdural haemorrhage (acute)(nontraumatic)  I62.1 Nontraumatic extradural haemorrhage  I62.9 Intracranial haemorrhage (nontraumatic), unspecified  I63 Cerebral infarction  I63.0 Cerebral infarction due to thrombosis of precerebral arteries  I63.1 Cerebral infarction due to embolism of precerebral arteries  I63.2 Cerebral infarction due to unspecified occlusion or stenosis of precerebral arteries  I63.3 Cerebral infarction due to thrombosis of cerebral arteries  I63.4 Cerebral infarction due to embolism of cerebral arteries  I63.5 Cerebral infarction due to unspecified occlusion or stenosis of cerebral arteries  I63.8 Other cerebral infarction  I63.9 Cerebral infarction, unspecified  I64 Stroke, not specified as haemorrhage or infarction |
| Major complications of anaesthesia | O74.0 Aspiration pneumonitis due to anaesthesia during labour and delivery  O74.1 Other pulmonary complications of anaesthesia during labour and delivery  O74.2 Cardiac complications of anaesthesia during labour and delivery  O74.3 Central nervous system complications of anaesthesia during labour and delivery  O74.9 Complication of anaesthesia during labour and delivery, unspecified  O89.0 Pulmonary complications of anaesthesia during the puerperium  O89.1 Cardiac complications of anaesthesia during the puerperium  O89.2 Central nervous system complications of anaesthesia during the puerperium  O29.0 Pulmonary complications of anaesthesia during pregnancy  O29.1 Cardiac complications of anaesthesia during pregnancy  O29.2 Central nervous system complications of anaesthesia during pregnancy  O29.6 Failed or difficult intubation during pregnancy  O29.3 Toxic reaction to local anaesthesia during pregnancy |
| Embolic event (pulmonary embolism, amniotic fluid embolism, septic embolism and air embolism) | O88 Obstetric embolism  O88.0 Obstetric air embolism  O88.1 Amniotic fluid embolism  O88.2 Obstetric blood-clot embolism  O88.3 Obstetric pyaemic and septic embolism  O88.8 Other obstetric embolism  I26 Pulmonary embolism  I26.0 Pulmonary embolism with mention of acute cor pulmonale  I26.9 Pulmonary embolism without mention of acute cor pulmonale |
| Shock | R57.0 Cardiogenic shock  R57.1 Hypovolaemic shock  R57.2 Septic shock  R57.8 Other shock  R57.9 Shock, unspecified  O75.1 Shock during or following labour and delivery  T80.5 Anaphylactic shock due to serum  T88.6 Anaphylactic shock due to adverse effect of correct drug or medicament properly administered  T78.2 Anaphylactic shock, unspecified  T78.0 Anaphylactic shock due to adverse food reaction  A48.3 Toxic shock syndrome |
| Sickle cell anaemia with crisis | D57.0 Sickle-cell anaemia with crisis |
| Status asthmaticus | J46 Status asthmaticus |
| Status epilepticus | G41 Status epilepticus  G41.0 Grand mal status epilepticus  G41.1 Petit mal status epilepticus  G41.2 Complex partial status epilepticus  G41.8 Other status epilepticus  G41.9 Status epilepticus, unspecified |
| Uterine rupture | O71.0 Rupture of uterus before onset of labour  O71.1 Rupture of uterus during labour |
| Eclampsia | O15 Eclampsia  O15.0 Eclampsia in pregnancy  O15.1 Eclampsia in labour  O15.2 Eclampsia in the puerperium  O15.9 Eclampsia, unspecified as to time period |
| Sepsis | O85 Puerperal sepsis  A40 Streptococcal sepsis  A40.0 Sepsis due to streptococcus, group A  A40.1 Sepsis due to streptococcus, group B  A40.2 Sepsis due to streptococcus, group D  A40.3 Sepsis due to Streptococcus pneumoniae  A40.8 Other streptococcal sepsis  A40.9 Streptococcal sepsis, unspecified  A41 Other sepsis  A41.0 Sepsis due to Staphylococcus aureus  A41.1 Sepsis due to other specified staphylococcus  A41.2 Sepsis due to unspecified staphylococcus  A41.3 Sepsis due to Haemophilus influenzae  A41.4 Sepsis due to anaerobes  A41.5 Sepsis due to other Gram-negative organisms  A41.8 Other specified sepsis  A41.9 Sepsis, unspecified (including septicaemia)  A32.7 Listerial sepsis |
| Cerebral venous thrombosis | O87.3 Cerebral venous thrombosis in the puerperium  I63.6 Cerebral infarction due to cerebral venous thrombosis, nonpyogenic  I67.6 Nonpyogenic thrombosis of intracranial venous system (including non-pyogenic thrombosis of cerebral vein and intracranial venous sinus) |
| Acute pancreatitis | K85 Acute pancreatitis  K85.0 Idiopathic acute pancreatitis  K85.1 Biliary acute pancreatitis  K85.2 Alcohol-induced acute pancreatitis  K85.3 Drug-induced acute pancreatitis  K85.8 Other acute pancreatitis  K85.9 Acute pancreatitis, unspecified  K86.3 Pseudocyst of pancreas |
| Rupture of aortic aneurysm or dissection of aorta | I71.0 Dissection of aorta [any part]  I71.1 Thoracic aortic aneurysm, ruptured  I71.3 Abdominal aortic aneurysm, ruptured  I71.5 Thoracoabdominal aortic aneurysm, ruptured  I71.8 Aortic aneurysm of unspecified site, ruptured  I72.2 Aneurysm and dissection of renal artery  I72.3 Aneurysm and dissection of iliac artery  I71.2 Thoracic aortic aneurysm, without mention of rupture  I71.4 Abdominal aortic aneurysm, without mention of rupture  I71.6 Thoracoabdominal aortic aneurysm, without mention of rupture  I71.9 Aortic aneurysm of unspecified site, without mention of rupture |
| Diabetic ketoacidosis | E10.0 Diabetes mellitus with coma (including hyperglycaemic coma NOS, diabetic coma with or without ketoacidosis, diabetic hyperosmolar coma, diabetic hypoglycaemic coma)  E10.1 Diabetes mellitus with ketoacidosis |
| **Morbid event category (OPCS-4 codes)** | **Codes** |
| Respiratory support | E85.1 Invasive ventilation (Includes endotracheal intermittent positive pressure ventilation) E85.2 Non-invasive ventilation NEC (Includes continuous positive airway pressure, intermittent positive pressure ventilation NEC, Negative pressure ventilation, Bilevel positive airway pressure, High flow continuous positive airway pressure)  E42.1 Permanent tracheostomy E42.2 Cricothyroidostomy E42.3 Temporary tracheostomy (includes tracheostomy NEC, Traccheostomy, Placement of tracheostomy tube) E42.8 Other specified (Exteriorisation of trachea) E42.9 Unspecified (Exteriorisation of trachea)  E85.6: Continuous positive airway pressure  E85.8: Other specified ventilation support  E85.9: Unspecified ventilation support  E89.9: Unspecified other respiratory support  E89.8: Other specified other respiratory support  Y73.3: Ventilatory support  Y73.1: Cardiopulmonary bypass  Y73.2: Extracorporeal circulation NEC |
| Surgical evacuation of the uterus following birth | R28.1 Curettage of delivered uterus  R28.8 Other specified (Instrumental removal of products of conception from delivered uterus) R28.9 Unspecified (Instrumental removal of products of conception from delivered uterus)  Q10 Curettage of uterus  Q10.1 Dilation of cervix uteri and curettage of products of conception from uterus  Q10.2 Curettage of products of conception from uterus NEC  Q10.3 Dilation of cervix uteri and curettage of uterus NEC  Q10.8 Other specified  Q10.9 Unspecified  Q11 Other evacuation of contents of uterus  Q11.1 Vacuum aspiration of products of conception from uterus NEC  Q11.5 Vacuum aspiration of products of conception from uterus using rigid cannula Q11.6 Vacuum aspiration of products of conception from uterus using flexible cannula  Q11.3 Evacuation of products of conception from uterus NEC |
| Dialysis | X40 Compensation for renal failure  X40.1 Renal dialysis  X40.2 Peritoneal dialysis NEC  X40.3 Haemodialysis NEC  X40.4 Haemofiltration X40.5 Automated peritoneal dialysis X40.6 Continuous ambulatory peritoneal dialysis X40.7 Haemoperfusion X40.8 Other specified X40.9 Unspecified X41.1 Insertion of ambulatory peritoneal dialysis catheter  X42.1 Insertion of temporary peritoneal dialysis catheter |
| Management of intra-abdominal or pelvic collection | T34.1 Open drainage of subphrenic abscess T34.2 Open drainage of pelvic abscess T34.3 Open drainage of abdominal abscess NEC  T45.1 Image controlled percutaneous drainage of subphrenic abscess T45.2 Image controlled percutaneous drainage of pelvic abscess T45.3 Image controlled percutaneous drainage of abdominal abscess NEC T45.4 Image controlled percutaneous drainage of lesion of abdominal cavity NEC  Y22 Drainage of organ NOC  Y22.1 Aspiration of haematoma of organ NOC  T34.8 Other specified (open drainage of peritoneum) T34.9 Unspecified (open drainage of peritoneum)  T46.8: Other specified other drainage of peritoneal cavity  T46.9: Unspecified other drainage of peritoneal cavity  T96.3: Debridement of soft tissue NEC  Y05.5: Debridement of organ NOC  Y22.8: Other specified drainage of organ NOC  Y22.9: Unspecified drainage of organ NOC  Y25.1: Suture of laceration of organ NOC  Y25.2: Resuture of organ NOC  Y32.1: Re-exploration of organ and surgical arrest of postoperative bleeding NOC  Y32.2: Re-exploration of organ and other repair of organ NOC  Y32.3: Re-exploration of organ and packing of organ NOC  Y32.8: Other specified re-exploration of organ NOC  Y32.9: Unspecified re-exploration of organ NOC  T30.1: Reopening of abdomen and re-exploration of intra-abdominal operation site and surgical arrest of postoperative bleeding  T30.2: Reopening of abdomen and re-exploration of intra-abdominal operation site NEC  T30.3: Reopening of abdomen NEC  H03.1: Drainage of abscess of appendix  H58.1: Drainage of ischiorectal abscess  H58.3: Drainage of perirectal abscess |
| Management of vulval or vaginal haematoma | P09.3 Evacuation of haematoma from vulva  P27.1 Evacuation of haematoma from vagina |
| Hysterectomy | Q07.4 Total abdominal hysterectomy NEC  Q07.5 Subtotal abdominal hysterectomy  R25.1 Caesarean hysterectomy |
| Interventional and surgical procedures to arrest major haemorrhage following birth | L70.2 Open embolisation of artery NEC  L70.3 Ligation of artery NEC  L71.3 Percutaneous transluminal embolisation of artery  L93.3 Ligation of vein NEC  L94.1 Percutaneous transluminal embolisation of vein  L99.5: Percutaneous transluminal occlusion of vein NEC  L53.1: Repair of iliac artery NEC  L53.8: Other specified other open operations on iliac artery  L53.9: Unspecified other open operations on iliac artery  L54.3: Arteriography of iliac artery  L66.3: Percutaneous transluminal occlusion of artery  L72.1: Arteriography NEC  L93.7: Repair of vein NEC  L97.4: Operations on artery NEC  L97.5: Operations on vein NEC  L97.4: Operations on artery NEC  L97.5: Operations on vein NEC  Y78.1: Arteriotomy approach to organ using image guidance with fluoroscopy  Y78.2: Arteriotomy approach to organ using image guidance with computed tomography  Y79.3: Transluminal approach to organ through femoral artery  Y78.8: Other specified arteriotomy approach to organ under image control  Y78.9: Unspecified arteriotomy approach to organ under image control  Y79.8: Other specified approach to organ through artery  Y79.9: Unspecified approach to organ through artery |
| Management of caesarean wound dehiscence | T28.3: Resuture of previous incision of anterior abdominal wall  T28.8: Other specified other repair of anterior abdominal wall  T28.9: Unspecified other repair of anterior abdominal wall |
| Repair of damage to bladder or urinary tract | M37.3: Repair of rupture of bladder  M37.8: Other specified other repair of bladder  M37.9: Unspecified other repair of bladder  M73.6: Urethroplasty NEC  M73.7: Repair of rupture of urethra NEC  M73.8: Other specified repair of urethra  M13.6: Percutaneous insertion of nephrostomy tube  M18.2: Excision of segment of ureter  M18.8: Other specified excision of ureter  M18.9: Unspecified excision of ureter  M19.1: Construction of ileal conduit  M19.2: Creation of urinary diversion to intestine NEC  M19.4: Cutaneous ureterostomy NEC  M20.1: Bilateral replantation of ureter  M20.2: Unilateral replantation of ureter  M20.3: Replantation of ureter after urinary diversion  M20.8: Other specified replantation of ureter  M20.9: Unspecified replantation of ureter  M21.1: Direct anastomosis of ureter to bladder  M21.2: Anastomosis of ureter to bladder using flap of bladder  M21.3: Ileal replacement of ureter  M21.4: Colonic replacement of ureter  M21.6: Ureteroureterostomy  M22.1: Suture of ureter  M22.2: Removal of ligature from ureter  M22.8: Other specified repair of ureter  M22.9: Unspecified repair of ureter  M27.4: Ureteroscopic insertion of ureteric stent  M27.7: Ureteroscopic dilation of ureter  M27.8: Other specified therapeutic ureteroscopic operations on ureter  M27.9: Unspecified therapeutic ureteroscopic operations on ureter  M29.4: Endoscopic dilation of ureter  M33.1: Percutaneous insertion of metallic stent into ureter  M33.2: Percutaneous insertion of plastic stent into ureter  M33.8: Other specified percutaneous ureteric stent procedures  M33.9: Unspecified percutaneous ureteric stent procedures  M73.4: Reconstruction of urethra  M73.8: Other specified repair of urethra  M73.9: Unspecified repair of urethra |
| Repair of damage to the intestine and management of intestinal obstruction | G58.1: Total jejunectomy and anastomosis of stomach to ileum  G58.2: Total jejunectomy and anastomosis of duodenum to ileum  G58.3: Total jejunectomy and anastomosis of duodenum to colon  G58.4: Partial jejunectomy and anastomosis of jejunum to ileum  G58.5: Partial jejunectomy and anastomosis of duodenum to colon  G58.8: Other specified excision of jejunum  G58.9: Unspecified excision of jejunum  G69.1: Ileectomy and anastomosis of stomach to ileum  G69.2: Ileectomy and anastomosis of duodenum to ileum  G69.3: Ileectomy and anastomosis of ileum to ileum  G69.4: Ileectomy and anastomosis of ileum to colon  G69.8: Other specified excision of ileum  G69.9: Unspecified excision of ileum  G78.4: Closure of perforation of ileum  G78.5: Exclusion of segment of ileum  G78.6: Open intubation of ileum  H06.1: Extended right hemicolectomy and end to end anastomosis  H06.2: Extended right hemicolectomy and anastomosis of ileum to colon  H06.9: Unspecified extended excision of right hemicolon  H07.1: Right hemicolectomy and end to end anastomosis of ileum to colon  H07.2: Right hemicolectomy and side to side anastomosis of ileum to transverse colon  H07.3: Right hemicolectomy and anastomosis NEC  H07.4: Right hemicolectomy and ileostomy HFQ  H07.5: Right hemicolectomy and end to side anastomosis  H07.8: Other specified other excision of right hemicolon  H07.9: Unspecified other excision of right hemicolon  H08.1: Transverse colectomy and end to end anastomosis  H08.2: Transverse colectomy and anastomosis of ileum to colon  H08.3: Transverse colectomy and anastomosis NEC  H08.4: Transverse colectomy and ileostomy HFQ  H08.5: Transverse colectomy and exteriorisation of bowel NEC  H08.6: Transverse colectomy and end to side anastomosis  H08.8: Other specified excision of transverse colon  H08.9: Unspecified excision of transverse colon  H09.1: Left hemicolectomy and end to end anastomosis of colon to rectum  H09.2: Left hemicolectomy and end to end anastomosis of colon to colon  H11.2: Colectomy and side to side anastomosis of ileum to colon NEC  H11.3: Colectomy and anastomosis NEC  H11.4: Colectomy and ileostomy NEC  H11.5: Colectomy and exteriorisation of bowel NEC  H11.6: Colectomy and end to side anastomosis NEC  H11.8: Other specified other excision of colon  H11.9: Unspecified other excision of colon  H29.1: Subtotal excision of colon and rectum and creation of colonic pouch and anastomosis of colon to anus  H29.2: Subtotal excision of colon and rectum and creation of colonic pouch NEC  H29.3: Subtotal excision of colon and creation of colonic pouch and anastomosis of colon to rectum  H29.4: Subtotal excision of colon and creation of colonic pouch NEC  H29.5: Subtotal excision of colon and anastomosis of colon to ileum  H29.8: Other specified subtotal excision of colon  H29.9: Unspecified subtotal excision of colon  H33.1: Abdominoperineal excision of rectum and end colostomy  H33.2: Proctectomy and anastomosis of colon to anus  H33.3: Anterior resection of rectum and anastomosis of colon to rectum using staples  H33.4: Anterior resection of rectum and anastomosis NEC  H33.5: Rectosigmoidectomy and closure of rectal stump and exteriorisation of bowel  H33.6: Anterior resection of rectum and exteriorisation of bowel  H33.7: Perineal resection of rectum HFQ  H33.8: Other specified excision of rectum  H33.9: Unspecified excision of rectum  T37.4: Repair of mesentery of small intestine  T38.4: Repair of mesentery of colon  H17.2: Open reduction of volvulus of caecum  H17.3: Open reduction of volvulus of sigmoid colon  H17.4: Open reduction of volvulus of colon NEC  H17.5: Open relief of strangulation of colon  H17.6: Open relief of obstruction of colon NEC  H15.8: Other specified other exteriorisation of colon  H15.9: Unspecified other exteriorisation of colon  H15.1: Loop colostomy  H15.2: End colostomy  H13.1: Bypass of colon by anastomosis of ileum to colon  H13.2: Bypass of colon by anastomosis of caecum to sigmoid colon  H13.3: Bypass of colon by anastomosis of transverse colon to sigmoid colon  H13.4: Bypass of colon by anastomosis of transverse colon to rectum  H13.5: Bypass of colon by anastomosis of colon to rectum NEC  H13.8: Other specified bypass of colon  H13.9: Unspecified bypass of colon  H10.1: Sigmoid colectomy and end to end anastomosis of ileum to rectum  H10.2: Sigmoid colectomy and anastomosis of colon to rectum  H10.3: Sigmoid colectomy and anastomosis NEC  H10.4: Sigmoid colectomy and ileostomy HFQ  H10.5: Sigmoid colectomy and exteriorisation of bowel NEC  H10.6: Sigmoid colectomy and end to side anastomosis  H10.8: Other specified excision of sigmoid colon  H10.9: Unspecified excision of sigmoid colon  H05.1: Total colectomy and anastomosis of ileum to rectum  H05.2: Total colectomy and ileostomy and creation of rectal fistula HFQ  H05.3: Total colectomy and ileostomy NEC  H05.8: Other specified total excision of colon  H05.9: Unspecified total excision of colon  G63.3: Closure of perforation of jejunum  G72.1: Anastomosis of ileum to caecum  G72.2: Anastomosis of ileum to transverse colon  G72.3: Anastomosis of ileum to colon NEC  G72.4: Anastomosis of ileum to rectum  G72.5: Anastomosis of ileum to anus and creation of pouch HFQ  G74.1: Creation of continent ileostomy  G74.2: Creation of temporary ileostomy  G74.3: Creation of defunctioning ileostomy  G76.2: Open relief of strangulation of ileum  G76.3: Open relief of obstruction of ileum NEC  T30.5: Packing of abdominal cavity |
| Management of acute coronary syndrome | K63.4: Coronary arteriography using two catheters  K63.5: Coronary arteriography using single catheter  K63.6: Coronary arteriography NEC  K63.8: Other specified contrast radiology of heart  K63.9: Unspecified contrast radiology of heart  K65.1: Catheterisation of combination of right and left side of heart NEC  K65.2: Catheterisation of right side of heart NEC  K65.3: Catheterisation of left side of heart NEC  K65.4: Catheterisation of left side of heart via atrial transeptal puncture  K65.8: Other specified catheterisation of heart  K65.9: Unspecified catheterisation of heart  K75.1: Percutaneous transluminal balloon angioplasty and insertion of 1-2 drug-eluting stents into coronary artery  K75.2: Percutaneous transluminal balloon angioplasty and insertion of 3 or more drug-eluting stents into coronary artery  K75.3: Percutaneous transluminal balloon angioplasty and insertion of 1-2 stents into coronary artery  K75.4: Percutaneous transluminal balloon angioplasty and insertion of 3 or more stents into coronary artery NEC  K75.8: Other specified percutaneous transluminal balloon angioplasty and insertion of stent into coronary artery  K75.9: Unspecified percutaneous transluminal balloon angioplasty and insertion of stent into coronary artery  L76.1: Endovascular placement of one metallic stent  L76.2: Endovascular placement of one plastic stent  L76.3: Endovascular placement of two metallic stents  L76.4: Endovascular placement of two plastic stents  L76.5: Endovascular placement of three or more metallic stents  L76.6: Endovascular placement of three or more plastic stents  L76.7: Endovascular placement of metallic stent with mechanical embolic protection  L76.8: Other specified endovascular placement of stent  L76.9: Unspecified endovascular placement of stent  L89.1: Endovascular placement of two drug-eluting stents  L89.2: Endovascular placement of two coated stents  L89.3: Endovascular placement of three or more drug-eluting stents  L89.4: Endovascular placement of three or more coated stents  L89.5: Endovascular placement of one drug-eluting stent  L89.6: Endovascular placement of one coated stent  L89.8: Other specified other endovascular placement of stent  L89.9: Unspecified other endovascular placement of stent |
| Interventional and surgical procedures to manage thromboembolism | L79.1: Insertion of filter into vena cava  L12.4: Open embolectomy of pulmonary artery  L13.1: Percutaneous transluminal embolectomy of pulmonary artery  L96.1: Percutaneous mechanical thromboembolectomy  L96.2: Percutaneous aspiration thromboembolectomy  L96.8: Other specified percutaneous removal of thrombus from vein  L96.9: Unspecified percutaneous removal of thrombus from vein  L99.3: Percutaneous transluminal venous thrombolysis with reconstruction  L99.4: Percutaneous transluminal venous thrombolysis NEC |
| Interventional and surgical procedures to manage major haemorrhage originating from the spleen | J69.1: Total excision of spleen and replantation of fragments of spleen  J69.2: Total splenectomy  J69.8: Other specified total excision of spleen  J69.9: Unspecified total excision of spleen  J70.1: Partial splenectomy  J72.2: Embolisation of spleen  J72.4: Repair of spleen  J72.5: Banding of spleen |
| Interventional procedures to treat haemorrhagic or ischaemic stroke | L33.1: Excision of aneurysm of cerebral artery  L33.2: Clipping of aneurysm of cerebral artery  L33.3: Ligation of aneurysm of cerebral artery NEC  L33.4: Obliteration of aneurysm of cerebral artery NEC  L33.8: Other specified operations on aneurysm of cerebral artery  L33.9: Unspecified operations on aneurysm of cerebral artery  L34.3: Open embolectomy of cerebral artery  L34.4: Open embolisation of cerebral artery  L35.1: Percutaneous transluminal embolisation of cerebral artery  L35.2: Arteriography of cerebral artery  L35.3: Percutaneous transluminal insertion of stent into cerebral artery  L35.4: Percutaneous transluminal embolectomy of cerebral artery  L35.8: Other specified transluminal operations on cerebral artery  L35.9: Unspecified transluminal operations on cerebral artery  L96.1: Percutaneous mechanical thromboembolectomy  L96.2: Percutaneous aspiration thromboembolectomy  O01.1: Percutaneous transluminal coil embolisation of small aneurysm of artery  O01.2: Percutaneous transluminal coil embolisation of medium aneurysm of artery  O01.3: Percutaneous transluminal coil embolisation of large aneurysm of artery  O01.4: Percutaneous transluminal coil embolisation of giant aneurysm of artery  O01.8: Other specified transluminal coil embolisation of aneurysm of artery  O01.9: Unspecified transluminal coil embolisation of aneurysm of artery  O02.1: Percutaneous transluminal balloon assisted coil embolisation of three or more aneurysms of artery  O02.2: Percutaneous transluminal balloon assisted coil embolisation of two aneurysms of artery  O02.3: Percutaneous transluminal balloon assisted coil embolisation of single aneurysm of artery  O02.8: Other specified transluminal balloon assisted coil embolisation of aneurysm of artery  O02.9: Unspecified transluminal balloon assisted coil embolisation of aneurysm of artery  O03.1: Percutaneous transluminal stent assisted coil embolisation of three or more aneurysms of artery  O03.2: Percutaneous transluminal stent assisted coil embolisation of two aneurysms of artery  O03.3: Percutaneous transluminal stent assisted coil embolisation of single aneurysm of artery  O03.4: Percutaneous transluminal flow diverting stent assisted coil embolisation of three or more aneurysms of artery  O03.5: Percutaneous transluminal flow diverting stent assisted coil embolisation of two aneurysms of artery  O03.6: Percutaneous transluminal flow diverting stent assisted coil embolisation of single aneurysm of artery  O03.8: Other specified transluminal stent assisted coil embolisation of aneurysm of artery  O03.9: Unspecified transluminal stent assisted coil embolisation of aneurysm of artery  O04.1: Percutaneous transluminal liquid polymer embolisation of aneurysm of artery  O04.2: Percutaneous transluminal stent assisted liquid polymer embolisation of aneurysm of artery  O04.3: Percutaneous transluminal flow diverting stent embolisation of aneurysm of artery  O04.8: Other specified other transluminal embolisation of aneurysm of artery  O04.9: Unspecified other transluminal embolisation of aneurysm of artery |
| Repair of aortic aneurysm rupture or aortic dissection | L18.1: Emergency replacement of aneurysmal segment of ascending aorta by anastomosis of aorta to aorta  L18.2: Emergency replacement of aneurysmal segment of thoracic aorta by anastomosis of aorta to aorta NEC  L18.3: Emergency replacement of aneurysmal segment of suprarenal abdominal aorta by anastomosis of aorta to aorta  L18.4: Emergency replacement of aneurysmal segment of infrarenal abdominal aorta by anastomosis of aorta to aorta  L18.5: Emergency replacement of aneurysmal segment of abdominal aorta by anastomosis of aorta to aorta NEC  L18.6: Emergency replacement of aneurysmal bifurcation of aorta by anastomosis of aorta to iliac artery  L18.8: Other specified emergency replacement of aneurysmal segment of aorta  L18.9: Unspecified emergency replacement of aneurysmal segment of aorta  L19.1: Replacement of aneurysmal segment of ascending aorta by anastomosis of aorta to aorta NEC  L19.2: Replacement of aneurysmal segment of thoracic aorta by anastomosis of aorta to aorta NEC  L19.3: Replacement of aneurysmal segment of suprarenal abdominal aorta by anastomosis of aorta to aorta NEC  L19.4: Replacement of aneurysmal segment of infrarenal abdominal aorta by anastomosis of aorta to aorta NEC  L19.5: Replacement of aneurysmal segment of abdominal aorta by anastomosis of aorta to aorta NEC  L19.6: Replacement of aneurysmal bifurcation of aorta by anastomosis of aorta to iliac artery NEC  L19.8: Other specified other replacement of aneurysmal segment of aorta  L19.9: Unspecified other replacement of aneurysmal segment of aorta  L26.5: Percutaneous transluminal insertion of stent into aorta  L26.6: Transluminal aortic stent graft with fenestration NEC  L26.7: Transluminal aortic branched stent graft NEC  L25.4: Operations on aneurysm of aorta NEC  L27.1: Endovascular insertion of stent graft for infrarenal abdominal aortic aneurysm  L27.2: Endovascular insertion of stent graft for suprarenal aortic aneurysmL27.3: Endovascular insertion of stent graft for thoracic aortic aneurysm  L27.4: Endovascular insertion of stent graft for aortic dissection in any position  L27.5: Endovascular insertion of stent graft for aortic aneurysm of bifurcation NEC  L27.6: Endovascular insertion of stent graft for aorto-uni-iliac aneurysm  L27.8: Other specified transluminal insertion of stent graft for aneurysmal segment of aorta  L27.9: Unspecified transluminal insertion of stent graft for aneurysmal segment of aorta  L28.1: Endovascular insertion of stent for infrarenal abdominal aortic aneurysm  L28.2: Endovascular insertion of stent for suprarenal aortic aneurysm  L28.3: Endovascular insertion of stent for thoracic aortic aneurysm  L28.4: Endovascular insertion of stent for aortic dissection in any position  L28.5: Endovascular insertion of stent for aortic aneurysm of bifurcation NEC  L28.6: Endovascular insertion of stent for aorto-uni-iliac aneurysm  L28.8: Other specified transluminal operations on aneurysmal segment of aorta  L28.9: Unspecified transluminal operations on aneurysmal segment of aorta  O20.1: Endovascular placement of one branched stent graft  O20.2: Endovascular placement of one fenestrated stent graft  O20.3: Endovascular placement of one stent graft NEC  O20.4: Endovascular placement of two stent grafts  O20.5: Endovascular placement of three or more stent grafts  O20.8: Other specified endovascular placement of stent graft  O20.9: Unspecified endovascular placement of stent graft |
| Surgical management of acute pancreatitis | J60.1: Drainage of pancreatic duct  J57.6: Pancreatic necrosectomy  J60.2: Open removal of calculus from pancreatic duct  J60.3: Insertion of T tube into pancreatic duct  J61.2: Drainage of cyst of pancreas into transposed jejunum  J61.3: Drainage of cyst of pancreas into jejunum NEC  J61.4: Drainage of cyst of pancreas NEC |

**Figure S1: DAG for causal relationship between ethnicity and SMM mediated by deprivation**

**
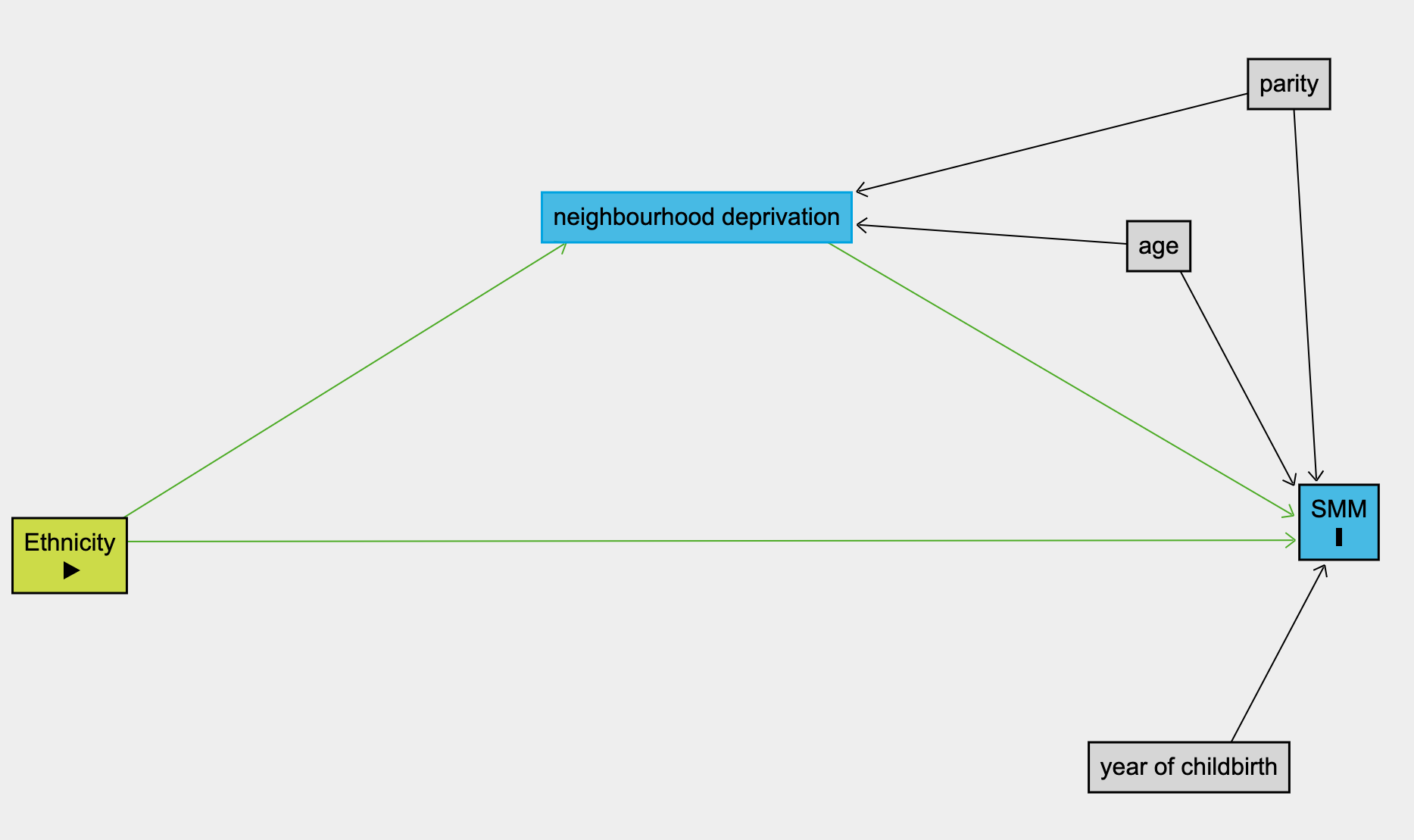
**

**Table S2: Definitions in the counterfactual framework (8)**

| **Effect** | **Counterfactual Definition** | **Interpretation** |
| --- | --- | --- |
| **Total Effect (TE)** | Ya−Y_a∗_Y_a_​−Y_a∗​_ | The total effect of exposure A (changing from a∗ to a) on the outcome Y. |
| **Pure Indirect Effect (NIE)** | Y_aM(a)_ – Y_aM(a*)_ | This effect is the contrast between the counterfactual outcome if the individual were exposed at A = a and the counterfactual outcome if the same individual were exposed at A = a*, with the mediator assuming whatever value it would have taken at the reference value of the exposure A = a* |
| **Natural Direct Effect (NDE)** | Y*_aM(a*)_*– Y*_a*,M(a*)_* | This effect is the contrast between the counterfactual outcome if the individual were exposed at A = a and the counterfactual outcome if the same individual were exposed at A = a*, with the mediator assuming whatever value it would have taken at the reference value of the exposure A = a*. |
| **Controlled Direct Effect (CDE)** | Y*_am_* – Y*_a*m_* | This effect is the contrast between the counterfactual outcome if the individual were exposed at A = a and the counterfactual outcome if the same individual were exposed at A = a*, with the mediator set to a fixed level M=*m*. |
| **Proportion Mediated** | NIE/TE | The proportion of the total effect that is mediated through the mediator M. |

*Y is the outcome, A is the exposure of interest and M is the potential mediator. For example, Y_am_ is denoted as the value of the outcome Y that would have been observed when exposure A is set to level a, and mediator M is set to level m. M_a_ is denoted as the value of the mediator M that would have been observed when the exposure A is set to level a.*

**Table S3. Characteristics of women by ethnic group. Number and proportion N (%).**

|  | **White** | **Black or Black British - Caribbean** | **Black or Black British - African** | **Asian or Asian British - Indian** | **Asian or Asian British - Pakistani** | **Asian or Asian British - Bangladeshi** | **Mixed** | **Other** | **Other Asian** | **Other Black** | **Total** |
| --- | --- | --- | --- | --- | --- | --- | --- | --- | --- | --- | --- |
|  | **N(%)** | **N(%)** | **N(%)** | **N(%)** | **N(%)** | **N(%)** | **N(%)** | **N(%)** | **N(%)** | **N(%)** | **N(%)** |
| N | 2,939,689 (76.6) | 37,046  (1.0) | 119,491 (3.1) | 135,491 (3.5) | 149,880 (3.9) | 56,646  (1.5) | 74,342 (1.9) | 190,615 (5.0) | 99,498 (2.6) | 36,458 (0.9) | 3,839,156 (100.0) |
| **Severe maternal morbidity** |  |  |  |  |  |  |  |  |  |  |  |
| No | 2,893,800 (98.4) | 36,068 (97.4) | 115,895 (97.0) | 132,658 (97.9) | 146,457 (97.7) | 54,993  (97.1) | 72,854 (98.0) | 186,501 (97.8) | 96,991 (97.5) | 35,425 (97.2) | 3,771,642 (98.2) |
| Yes | 45,889 (1.6) | 978  (2.6) | 3,596 (3.0) | 2,833 (2.1) | 3,423  (2.3) | 1,653  (2.9) | 1,488  (2.0) | 4,114 (2.2) | 2,507 (2.5) | 1,033 (2.8) | 67,514 (1.8) |
| **Index of Multiple Deprivation** |  |  |  |  |  |  |  |  |  |  |  |
| Most deprived 20 % | 665,971 (22.7) | 16,363 (44.2) | 56,529 (47.3) | 28,995 (21.4) | 76,829 (51.3) | 29,469  (52.0) | 24,810 (33.4) | 60,266 (31.6) | 26,605 (26.7) | 15,569 (42.7) | 1,001,406 (26.1) |
| More deprived 20-40% | 623,508 (21.2) | 11,260 (30.4) | 33,764 (28.3) | 39,187 (28.9) | 37,538 (25.0) | 16,301  (28.8) | 18,537 (24.9) | 49,783 (26.1) | 28,187 (28.3) | 11,312 (31.0) | 869,377 (22.6) |
| Less deprived 40-60% | 590,306 (20.1) | 5,304  (14.3) | 15,279 (12.8) | 29,243 (21.6) | 18,864 (12.6) | 6,208  (11.0) | 12,763 (17.2) | 34,359 (18.0) | 20,346 (20.4) | 5,177 (14.2) | 737,849 (19.2) |
| Less deprived 60-80% | 554,122 (18.8) | 2,602  (7.0) | 8,403 (7.0) | 20,346 (15.0) | 10,219  (6.8) | 2,968  (5.2) | 10,025 (13.5) | 25,299 (13.3) | 13,833 (13.9) | 2,652 (7.3) | 650,469 (16.9) |
| Least deprived 80-100% | 505,782 (17.2) | 1,517  (4.1) | 5,516 (4.6) | 17,720 (13.1) | 6,430  (4.3) | 1,700  (3.0) | 8,207 (11.0) | 20,908 (11.0) | 10,527 (10.6) | 1,748 (4.8) | 580,055 (15.1) |

**Table S3. (Continued)**

|  | **White** | **Black or Black British - Caribbean** | **Black or Black British - African** | **Asian or Asian British - Indian** | **Asian or Asian British - Pakistani** | **Asian or Asian British - Bangladeshi** | **Mixed** | **Other** | **Other Asian** | **Other Black** | **Total** |
| --- | --- | --- | --- | --- | --- | --- | --- | --- | --- | --- | --- |
|  | **N(%)** | **N(%)** | **N(%)** | **N(%)** | **N(%)** | **N(%)** | **N(%)** | **N(%)** | **N(%)** | **N(%)** | **N(%)** |
| **Age group, years** |  |  |  |  |  |  |  |  |  |  |  |
| <20 | 104,774 (3.6) | 1,318  (3.6) | 1,389 (1.2) | 353  (0.3) | 1,194  (0.8) | 464  (0.8) | 3,377  (4.5) | 4,020 (2.1) | 809 (0.8) | 930 (2.6) | 118,628 (3.1) |
| 20-25 | 435,355 (14.8) | 5,566  (15.0) | 10,343 (8.7) | 7,705 (5.7) | 17,240 (11.5) | 6,573  (11.6) | 11,712 (15.8) | 20,217 (10.6) | 8,724 (8.8) | 4,465 (12.2) | 527,900 (13.8) |
| 25-30 | 793,815 (27.0) | 8,989  (24.3) | 29,522 (24.7) | 34,658 (25.6) | 46,977 (31.3) | 17,960  (31.7) | 19,152 (25.8) | 45,933 (24.1) | 25,946 (26.1) | 9,318 (25.6) | 1,032,270 (26.9) |
| 30-35 | 936,178 (31.8) | 10,372 (28.0) | 38,888 (32.5) | 56,065 (41.4) | 49,268 (32.9) | 18,709  (33.0) | 22,250 (29.9) | 64,052 (33.6) | 35,498 (35.7) | 11,049 (30.3) | 1,242,329 (32.4) |
| 35-40 | 538,841 (18.3) | 7,813  (21.1) | 28,608 (23.9) | 30,564 (22.6) | 27,883 (18.6) | 10,444  (18.4) | 14,035 (18.9) | 43,830 (23.0) | 22,202 (22.3) | 7,880 (21.6) | 732,100 (19.1) |
| >40 | 130,726 (4.4) | 2,988  (8.1) | 10,741 (9.0) | 6,146 (4.5) | 7,318  (4.9) | 2,496  (4.4) | 3,816  (5.1) | 12,563 (6.6) | 6,319 (6.4) | 2,816 (7.7) | 185,929 (4.8) |
| **Parity** |  |  |  |  |  |  |  |  |  |  |  |
| Multiparous | 1,718,147 (58.4) | 24,427 (65.9) | 83,906 (70.2) | 81,879 (60.4) | 106,219 (70.9) | 42,423  (74.9) | 43,568 (58.6) | 110,345 (57.9) | 60,613 (60.9) | 24,308 (66.7) | 2,295,835 (59.8) |
| Primiparous | 1,221,542 (41.6) | 12,619 (34.1) | 35,585 (29.8) | 53,612 (39.6) | 43,661 (29.1) | 14,223  (25.1) | 30,774 (41.4) | 80,270 (42.1) | 38,885 (39.1) | 12,150 (33.3) | 1,543,321 (40.2) |

**Table S3. (Continued)**

|  | **White** | **Black or Black British - Caribbean** | **Black or Black British - African** | **Asian or Asian British - Indian** | **Asian or Asian British - Pakistani** | **Asian or Asian British - Bangladeshi** | **Mixed** | **Other** | **Other Asian** | **Other Black** | **Total** |
| --- | --- | --- | --- | --- | --- | --- | --- | --- | --- | --- | --- |
|  | **N(%)** | **N(%)** | **N(%)** | **N(%)** | **N(%)** | **N(%)** | **N(%)** | **N(%)** | **N(%)** | **N(%)** | **N(%)** |
| **Year** |  |  |  |  |  |  |  |  |  |  |  |
| 2013 | 335,738 (11.4) | 4,400  (11.9) | 13,372 (11.2) | 14,440 (10.7) | 17,285 (11.5) | 5,781  (10.2) | 6,797  (9.1) | 18,152 (9.5) | 9,748 (9.8) | 4,113 (11.3) | 429,826 (11.2) |
| 2014 | 326,453 (11.1) | 3,952  (10.7) | 12,280 (10.3) | 13,876 (10.2) | 16,583 (11.1) | 5,776  (10.2) | 6,876  (9.2) | 18,725 (9.8) | 9,875 (9.9) | 3,832 (10.5) | 418,228 (10.9) |
| 2015 | 311,742 (10.6) | 3,881  (10.5) | 11,959 (10.0) | 12,894 (9.5) | 15,507 (10.3) | 5,509  (9.7) | 6,836  (9.2) | 18,784 (9.9) | 9,459 (9.5) | 3,661 (10.0) | 400,232 (10.4) |
| 2016 | 301,111 (10.2) | 3,850  (10.4) | 12,160 (10.2) | 12,583 (9.3) | 15,487 (10.3) | 6,139  (10.8) | 7,013  (9.4) | 19,351 (10.2) | 9,472 (9.5) | 3,849 (10.6) | 391,015 (10.2) |
| 2017 | 284,370 (9.7) | 3,685  (9.9) | 11,427 (9.6) | 12,252 (9.0) | 14,311  (9.5) | 5,596  (9.9) | 7,025  (9.4) | 18,801 (9.9) | 9,178 (9.2) | 3,498 (9.6) | 370,143 (9.6) |
| 2018 | 269,995 (9.2) | 3,502  (9.5) | 10,538 (8.8) | 11,475 (8.5) | 12,852  (8.6) | 5,051  (8.9) | 6,979  (9.4) | 18,622 (9.8) | 8,533 (8.6) | 3,466 (9.5) | 351,013 (9.1) |
| 2019 | 262,794 (8.9) | 3,329  (9.0) | 10,138 (8.5) | 11,465 (8.5) | 13,019  (8.7) | 4,939  (8.7) | 7,129  (9.6) | 18,007 (9.4) | 8,737 (8.8) | 3,321 (9.1) | 342,878 (8.9) |
| 2020 | 254,702 (8.7) | 3,119  (8.4) | 10,210 (8.5) | 12,456 (9.2) | 13,081  (8.7) | 4,993  (8.8) | 7,071  (9.5) | 18,267 (9.6) | 9,509 (9.6) | 3,058 (8.4) | 336,466 (8.8) |
| 2021 | 276,850 (9.4) | 3,179  (8.6) | 11,008 (9.2) | 14,523 (10.7) | 13,480  (9.0) | 5,558  (9.8) | 8,086 (10.9) | 18,557 (9.7) | 10,734 (10.8) | 3,260 (8.9) | 365,235 (9.5) |
| 2022 | 259,229 (8.8) | 3,381  (9.1) | 13,071 (10.9) | 15,475 (11.4) | 14,852  (9.9) | 5,841  (10.3) | 8,572 (11.5) | 19,066 (10.0) | 11,653 (11.7) | 3,536 (9.7) | 354,676 (9.2) |
| 2023 | 56,705 (1.9) | 768  (2.1) | 3,328 (2.8) | 4,052 (3.0) | 3,423  (2.3) | 1,463  (2.6) | 1,958  (2.6) | 4,283 (2.2) | 2,600 (2.6) | 864 (2.4) | 79,444 (2.1) |

Abbreviations: SMM=Severe maternal morbidity, IMD = Index of Multiple Deprivation

**Figure S2:** **Flow chart showing the identification of the cohort**

**
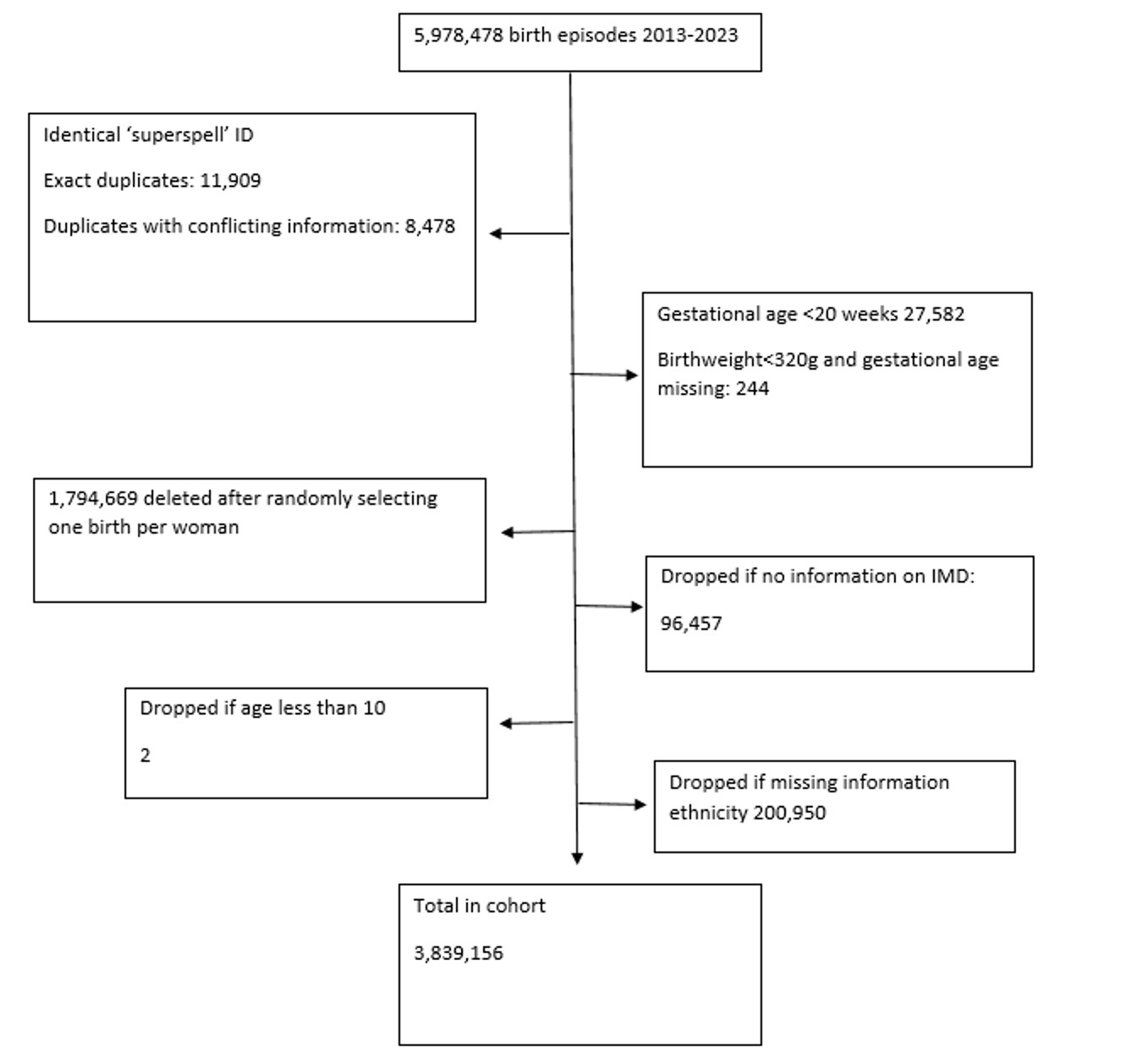
**

**Table S4: Number (N) and proportion (%) of severe maternal morbidity cases (SMM) by ethnicity and Index of Multiple Deprivation (IMD) quintile.**

| **Deprivation Quintile** | **White** | **Black or Black British - Caribbean** | **Black or Black British - African** | **Asian or Asian British - Indian** | **Asian or Asian British - Pakistani** | **Asian or Asian British - Bangladeshi** | **Mixed** | **Other** | **Other Asian** | **Other Black** |
| --- | --- | --- | --- | --- | --- | --- | --- | --- | --- | --- |
|  | **SMM/Total**  **N(%)** | **SMM/Total**  **N(%)** | **SMM/Total**  **N(%)** | **SMM/Total**  **N(%)** | **SMM/Total**  **N(%)** | **SMM/Total**  **N(%)** | **SMM/Total**  **N(%)** | **SMM/Total**  **N(%)** | **SMM/Total**  **N(%)** | **SMM/Total**  **N(%)** |
| **Most deprived 20%** | 10,757 / 665,971  (1.6) | 414 / 16,363  (2.5) | 1,759 / 56,529  (3.1) | 658 / 28,995 (2.3) | 1,777 / 76,829  (2.3) | 803 / 29,469 (2.7) | 501 / 24,810 (2.0) | 1,261 / 60,266 (2.1) | 677 / 26,605 (2.5) | 445 / 15,569  (2.9) |
| **More deprived 20-40%** | 10,051 / 623,508  (1.6) | 324 / 11,260  (2.9) | 979 / 33,764 (2.9) | 877 / 39,187 (2.2) | 869 / 37,538 (2.3) | 542 / 16,301 (3.3) | 411 / 18,537 (2.2) | 1,175 / 49,783 (2.4) | 770 / 28,187 (2.7) | 304 / 11,312  (2.7) |
| **Less deprived 40-60%** | 9,300 / 590,306  (1.6) | 136 / 5,304 (2.6) | 470 / 15,279 (3.1) | 580 / 29,243 (2.0) | 408 / 18,864 (2.2) | 179 / 6,208 (2.9) | 243 / 12,763 (1.9) | 772 / 34,359 (2.3) | 524 / 20,346 (2.6) | 155 / 5,177  (3.0) |
| **Less deprived 60-80%** | 8,478 / 554,122 (1.5) | 64 / 2,602 (2.5) | 250 / 8,403 (3.0) | 410 / 20,346 (2.0) | 237 / 10,219 (2.3) | 76 / 2,968 (2.6) | 194 / 10,025 (1.9) | 508 / 25,299 (2.0) | 300 / 13,833 (2.2) | 76 / 2,652  (2.9) |
| **Least deprived 80-100%** | 7,303 / 505,782  (1.4) | 40 / 1,517 (2.6) | 138 / 5,516 (2.5) | 308 / 17,720 (1.7) | 132 / 6,430 (2.1) | 53 / 1,700 (3.1) | 139 / 8,207 (1.7) | 398 / 20,908 (1.9) | 236 / 10,527 (2.2) | 53 / 1,748  (3.0) |
| **Total** | 45,889 / 2,939,689  (1.6) | 978 /  37,046  (2.6) | 3,596 /  119,491  (3.0) | 2,833 / 135,491  (2.1) | 3,423 / 149,880  (2.3) | 1,653 / 56,646  (2.9) | 1,488 /  74,342  (2.0) | 4,114 / 190,615  (2.2) | 2,507 /  99,498  (2.5) | 1,033 /  36,458  (2.8) |

**Table S5: Adjusted predictive probabilities and their 95% confidence interval (CI) of ethnic groups stratified by IMD compared to White least deprived women (Model 1). Adjusting for age, parity and year of childbirth**

|  | Probability (95% CI) |
| --- | --- |
| White |  |
| Q1 (least deprived) | 1.41  (1.37–1.44) |
| Q2 | 1.48  (1.45–1.52) |
| Q3 | 1.56  (1.52–1.59) |
| Q4 | 1.64  (1.60–1.67) |
| Q5 (most deprived) | 1.70  (1.66–1.73) |
| Black or Black British Caribbean |  |
| Q1 | 1.44  (1.40–1.47) |
| Q2 | 2.50  (1.72–3.27) |
| Q3 | 2.31  (1.74–2.87) |
| Q4 | 2.52  (2.10–2.95) |
| Q5 | 2.96  (2.63–3.29) |
| Black or Black British African |  |
| Q1 | 2.79  (2.52–3.06) |
| Q2 | 2.85  (2.50–3.21) |
| Q3 | 2.99  (2.72–3.27) |
| Q4 | 2.94  (2.76–3.13) |
| Q5 | 3.32  (3.16–3.48) |
| Asian or Asian British Indian |  |
| Q1 | 1.72  (1.53–1.91) |
| Q2 | 1.94  (1.75–2.13) |
| Q3 | 1.93  (1.77–2.09) |
| Q4 | 2.23  (2.08–2.38) |
| Q5 | 2.32  (2.14–2.50) |

**Table S5: (Continued)**

|  | Probability (95% CI) |
| --- | --- |
| Asian or Asian British Pakistani |  |
| Q1 | 2.05  (1.70–2.40) |
| Q2 | 2.32  (2.02–2.62) |
| Q3 | 2.25  (2.03–2.47) |
| Q4 | 2.45  (2.29–2.62) |
| Q5 | 2.47  (2.35–2.60) |
| Asian or Asian British Bangladeshi |  |
| Q1 | 3.01  (2.20–3.82) |
| Q2 | 2.46  (1.90–3.01) |
| Q3 | 2.87  (2.44–3.29) |
| Q4 | 3.33  (3.04–3.62) |
| Q5 | 3.05  (2.83–3.27) |
| Mixed |  |
| Q1 | 1.57  (1.31–1.84) |
| Q2 | 1.77  (1.52–2.02) |
| Q3 | 1.78  (1.56–2.01) |
| Q4 | 2.14  (1.93–2.34) |
| Q5 | 2.07  (1.88–2.25) |
| Other |  |
| Q1 | 1.80  (1.62–1.98) |
| Q2 | 1.88  (1.72–2.05) |
| Q3 | 2.14  (1.98–2.29) |
| Q4 | 2.31  (2.18–2.44) |
| Q5 | 2.17  (2.04–2.29) |

**Table S5: (Continued)**

|  | Probability (95% CI) |
| --- | --- |
| Other Asian |  |
| Q1 | 2.12  (1.85–2.39) |
| Q2 | 2.03  (1.80–2.26) |
| Q3 | 2.49  (2.27–2.70) |
| Q4 | 2.71  (2.51–2.90) |
| Q5 | 2.59  (2.39–2.79) |
| Other Black |  |
| Q1 | 2.81  (2.05–3.57) |
| Q2 | 2.68  (2.08–3.29) |
| Q3 | 2.88  (2.43–3.34) |
| Q4 | 2.78  (2.47–3.10) |
| Q5 | 3.08  (2.79–3.37) |

Abbreviations: RR = risk ratio, CI=confidence interval, Q1-Q5= quartile 1 to 5

**Table S6: Control direct effects (CDE) where the mediator=0 (a hypothetical scenario where nobody experiences deprivation) and the mediator=1 (a hypothetical scenario where everybody experiences deprivation) in the association between ethnicity and SMM, adjusting for age, parity, pre-existing medical conditions and year of childbirth**

| **Ethnicity** | **Mediator = 0**  **CDE (95%CI)** | **Mediator = 1**  **CDE (95% CI)** |
| --- | --- | --- |
| **Black or Black British - Caribbean** | 1.74 [1.28, 2.36] | 1.62 [1.47, 1.78] |
| **Black or Black British - African** | 1.69 [1.43, 2.00] | 1.91 [1.81, 2.00] |
| **Asian or Asian British - Indian** | 1.16 [1.04, 1.30] | 1.30 [1.20, 1.41] |
| **Asian or Asian British - Pakistani** | 1.44 [1.22, 1.71] | 1.44 [1.37, 1.51] |
| **Asian or Asian British - Bangladeshi** | 2.12 [1.63, 2.77] | 1.79 [1.66, 1.92] |
| **Mixed** | 1.08 [0.92, 1.28] | 1.20 [1.10, 1.31] |
| **Other** | 1.24 [1.12, 1.37] | 1.30 [1.23, 1.38] |
| **Other Asian** | 1.45 [1.27, 1.64] | 1.47 [1.36, 1.59] |
| **Other Black** | 1.96 [1.50, 2.55] | 1.79 [1.63, 1.96] |

Abbreviations: SMM= severe maternal morbidity, CI=confidence interval

References

1. Annie Herbert, Linda Wijlaars, Ania Zylbersztejn, David Cromwell, Pia Hardelid, Data Resource Profile: Hospital Episode Statistics Admitted Patient Care (HES APC), International Journal of Epidemiology, Volume 46, Issue 4, August 2017, Pages 1093–1093i, https://doi.org/10.1093/ije/dyx015
2. Haines N. Births in England and Wales: 2017 [Internet]. Births in England and Wales - Office for National Statistics. Office for National Statistics; 2018 (accessed June 2, 2022). Available from: <https://www.ons.gov.uk/peoplepopulationandcommunity/birthsdeathsandmarriages/livebirths/bulletins/birthsummarytablesenglandandwales/2017>
3. NHS Digital. NHS data dictionary: ethnic category code 2001. https://www.datadictionary.nhs.uk/data_dictionary/attributes/e/ end/ethnic_category_code_2001_de.asp (accessed June 2, 2023)
4. Gallimore ID, Matthews RJ, Page GL. MBRRACE-UK perinatal mortality surveillance, UK perinatal deaths of babies born in 2022: State of the nation report. 2024
5. Office for National Statistics. Understanding consistency of ethnicity data recorded in health-related administrative datasets in England: 2011 to 2021.
6. D’Arcy RS. Investigating the Health and Care Needs of Pregnant Women with Multiple Long-Term Conditions [PhD thesis (awaiting publication)] 2024
7. Nair M, Kurinczuk JJ, Knight M. Establishing a national maternal morbidity outcome indicator in England: a population-based study using routine hospital data. PLoS One. 2016 Apr 7;11(4):e0153370
8. Richiardi L, Bellocco R, Zugna D. Mediation analysis in epidemiology: methods, interpretation and bias. International journal of epidemiology. 2013 Oct 1;42(5):1511-9.
